# Open focused microwave-assisted sample preparation for rapid total and mercury species determination in environmental solid samples

**DOI:** 10.1155/S1463924698000145

**Published:** 1998

**Authors:** C. M. Tseng, H. Garraud, D. Amouroux, O. F. X. Donard, A. de Diego

**Affiliations:** 1 Laboratoire de Chimie Bio-Inorganique et Environnement EP CNRS 132 Université de Pau et des Pays de l'Adour Hélioparc Pau 64000 France; 2 On leave from the Department of Analytical Chemistry University of the Basque Country 644 P.K. Bilbao 48080 Spain

## Abstract

This paper describes rapid, simple microwave-assisted leaching/ digestion procedures for total and mercury species determination in sediment samples and biomaterials. An open focused microwave system allowed the sample preparation time to be dramatically reduced to only 24 min when a power of 40-80 W was applied. Quantitative leaching of methylmercury from sediments by HNO_3_ solution and complete dissolution of biomaterials by an alkaline solution, such as 25% TMAH solution, were obtained. Methylmercury compounds were kept intact without decomposition or losses by evaporation. Quantitative recoveries of total mercury were achieved with a two-step microwave attack using a combination of HNO_3_ and H_2_0_2_ solutions as extractant. The whole pretreatment procedure only takes 15 min, which can be further shortened by an automated robust operation with an open focused system. These analytical procedures were validated by the analysis of environmental certified reference materials. The results confirm that the open focused microwave technique is a promising tool for solid sample preparation in analytical and environmental chemistry.


Journal of Automatic Chemistry, Vol. 20, No. 4 (July–August 1998) pp. 99–108

### Open focused microwave-assisted sample preparation for rapid total and mercury species determination in environmental solid samples

**C. M. Tseng, H. Garraud, D. Amouroux, O. F. X. Donard***

*Laboratoire de Chimie Bio-Inorganique et Environnement, EP CNRS 132, Université de Pau et des Pays de l'Adour, Hélioparc, 64000 Pau, France*

**and A. de Diego**

*On leave from the Department of Analytical Chemistry, University of the Basque Country, 644 P.K., 48080 Bilbao, Spain*

*This paper describes rapid, simple microwave-assisted leaching/digestion procedures for total and mercury species determination in sediment samples and biomaterials. An open focused microwave system allowed the sample preparation time to be dramatically reduced to only 2–4 min when a power of 40–80 W was applied. Quantitative leaching of methylmercury from sediments by $HNO_3$ solution and complete dissolution of biomaterials by an alkaline solution, such as 25% TMAH solution, were obtained. Methylmercury compounds were kept intact without decomposition or losses by evaporation. Quantitative recoveries of total mercury were achieved with a two-step microwave attack using a combination of $HNO_3$ and $H_2O_2$ solutions as extractant. The whole pretreatment procedure only takes 15 min, which can be further shortened by an automated robust operation with an open focused system. These analytical procedures were validated by the analysis of environmental certified reference materials. The results confirm that the open focused microwave technique is a promising tool for solid sample preparation in analytical and environmental chemistry.*

### Introduction

Sample preparation is one of the most crucial steps in trace element analysis and frequently controls the quality of the final results obtained [1, 2]. Environmental solid samples are generally made into a solution with wet digestion methods and analysed by compatible instrumental techniques, e.g. atomic absorption spectrometry (AAS), inductively coupled plasma coupled to atomic emission spectrometry (ICP-AES) or ICP-mass spectrometry (ICP-MS). Most of the conventional digestion procedures are not only laborious and time-consuming, but also lack sufficient efficiency and reliability. As a well-known example, hot plate digestion techniques with conductive heating, now used widely, easily lead to non-reproducible results. Other extraction methods, such as sonication, distillation or soxhlet extraction, also have the above drawbacks, even though reliable results are usually achieved. In the case of mercury speciation analysis, solid sample preparation by acid or alkaline extraction with different heating sources (sonication, stream distillation, etc.) requires from 2 to 24 h for complete recovery of the target analytes [3–10]. Innovative techniques such as supercritical fluid extraction (SFE) [11–13] and microwave-assisted extraction (MAE) [14–16] have been recently developed and are a substantial advance. However, SFE potentially has technological limitations and shows insufficient extraction efficiency, usually depending on sample matrix and analyte polarity. Moreover, the expensive equipment required increases the cost of the analysis and the extraction step still takes 20–50 min.

The main advantages of the microwave-assisted extraction technique are absence of inertia, rapidity of heating, reduction of extraction time, better reproducibility and reliability, ease of automation, and good ability for selective leaching and total digestion in a wide array of sample matrices [17]. Thus, the application of this technique to sample preparation has been widely investigated in various fields of the environmental and analytical chemistry since it was first applied in 1975 [18]. Two different approaches in microwave extraction procedures are the use of a closed system (pressurized with a closed vessel) or an open system (non-pressurized with an open vessel). They have different characteristics and applications, as shown in table 1 [16]. Nevertheless, for organometallic speciation analysis, open microwave technology based on focused microwaves is preferred to a closed microwave system, because better stability of the target compounds is achieved, due to the milder extraction conditions supplied (20–60 W, compared to 1000 W typically used in closed system); and better reproducibility is obtained, owing to a perfect control of the microwave energy, precisely focused on the sample. Essential parameters such as extraction medium, applied power, exposure time and sample size must be, however, fully optimized in terms of stability and extraction efficiency of the target analytes to set the optimum extraction conditions for further routine analysis [14–16, 19, 20].

This paper presents microwave-assisted leaching/digestion protocols for total and mercury speciation analysis in environmental solid samples, such as sediments and biological tissues, using an open and low-power focused microwave system (301 PROLABO). Total mercury in sediments was determined by flow injection sample introduction followed by ICP-MS detection, after two-step microwave-assisted acid digestion with concentrated $HNO_3$ and $H_2O_2$. Mercury species, such as methyl- and inorganic mercury, were analysed in both sediments and biological tissues by an automated on-line system

* Correspondence to Dr Donard.

0142-0453/98 $12·00 © 1998 Taylor & Francis Ltd

*Table 1. Comparison of the features between closed and open microwave systems for organometal speciation analysis.*

| Items | Multimode microwave closed system | Focused microwave open system |
|---|---|---|
| Easy to handle | Moderate[1] | Excellent |
| Optimization | Good[2] | Excellent |
| Temperature control | Good (only measured in one tube) | Excellent (independent in each tube) |
| Microwave control | Moderate (multiple reflection in the cavity) | Excellent (microwave focused on the sample) |
| Automatic addition of reagent | No | Yes (up to 4) |
| Amount of sample | <0.2 g | 0.1 to 5 g |
| Total digestion | Excellent | Excellent |
| Speciation analysis | Good | Excellent |
| Throughput | Excellent (up to 10 or 12 samples) | Good (up to six samples) |
| Safety | Good | Excellent |

1. Take into account the operations needed to handle the microwave tubes and to recover the sample after the digestion (cooling the tube and getting down the pressure inside).

2. Microwave conditions (temperature, pressure, time) are dependent on the number of samples exposed at the same time.

combining derivatization by ethylation or hydride generation, cryogenic trapping, gas chromatography and quartz furnace atomic absorption spectrometric detection (D-CT-GC-QFAAS), after microwave-assisted acid leaching/alkaline digestion with nitric acid solution or alkaline solutions, such as TMAH (tetramethylammonium hydroxide) or KOH-methanolic solution, respectively. The proposed methods were validated by analysis of four certified reference sediments and three certified reference biological materials. The results obtained are in good agreement with the certified values.

### Materials and methods

### Instrumentation

*Microwave system:* Sample extraction was carried out by a single-mode open focused microwave digestor 301 (PROLABO, France) with a frequency of 2450 MHz and a maximum power setting of 200 W, equipped with a TX 32 programmer. The power system provides a precise and continuous microwave emission from 10 to 200 W, in increments of 10 W, and allows a change of exposure time from 1 to 99 min in steps of 1 min. Both parameters are set by a TX 32 programmer. The microwave energy from the magnetron is delivered with high reproducibility and is focused on the sample. Thus higher heating efficiency is obtained. This single-mode microwave device was initially designed for sample extraction at atmospheric pressure with an open vessel (figure 1). A reflux system and an Aspivap fume treatment system (PROLABO, France) were used, to avoid possible losses of analytes and the escape of acid fumes generated during the extraction.

*Flow injection-inductively coupled plasma-mass spectrometry:* The FI-ICP-MS hyphenation includes a FIAS-200 FI

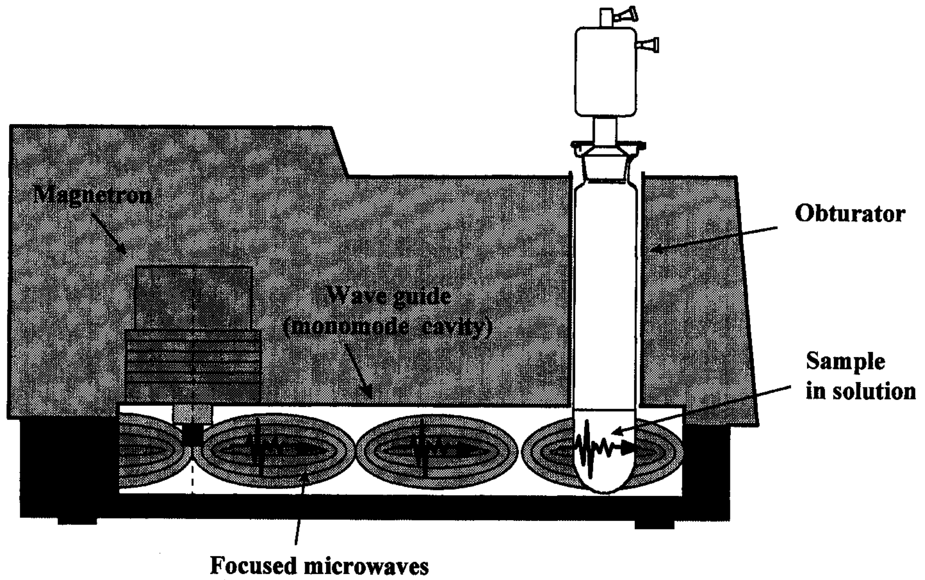

*Figure 1. Open focused microwave system (PROLABO, model 301).*

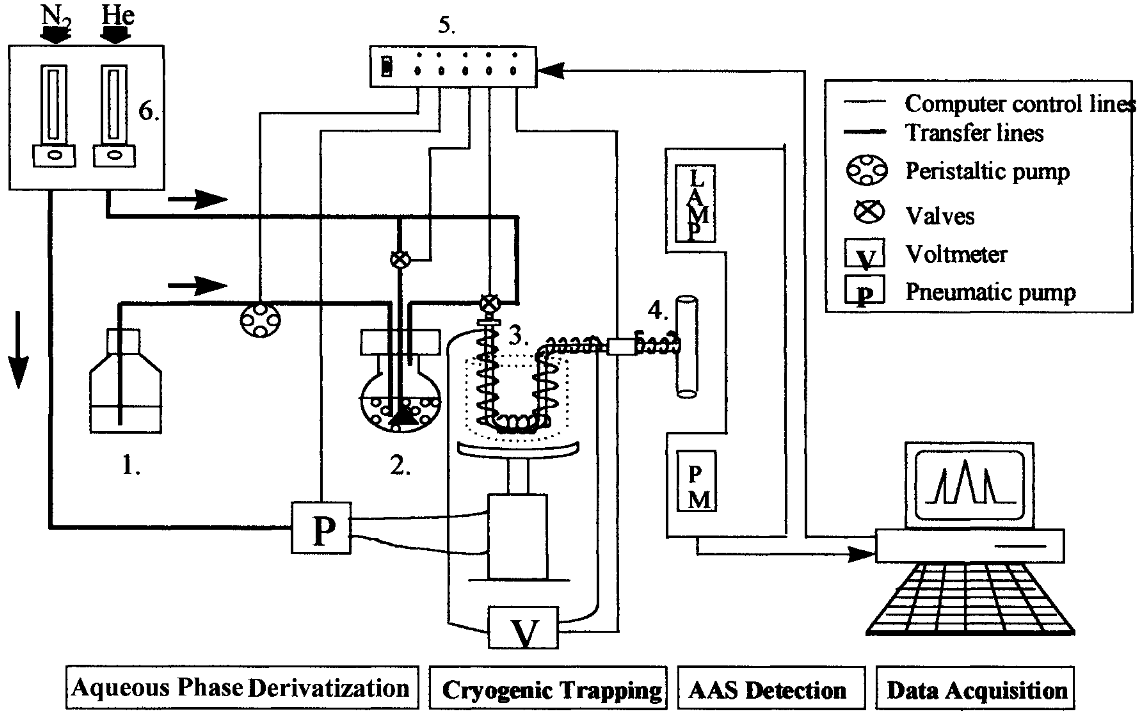

*Figure 2. Schematic diagram of the hyphenated system combining derivatization by hydride generation or ethylation, cryogenic trapping, gas chromatography and quartz furnace atomic spectroscopy: 1, reagent vessel; 2, reaction vessel; 3, cryogenic trap and GC column; 4, quartz furnace; 5, control panel; 6, flowmeters (adapted from Journal of Atomic Analytical Spectrometry, 1997,* ***12****, 629–635).*

system (Perkin-Elmer, Germany) and an ELAN 5000 ICP-MS instrument (Perkin-Elmer, Germany) and it has been used for total mercury analysis. The FI system includes two peristaltic pumps and an injection valve, fully controlled by the ELAN software. The ELAN 5000 ICP-MS instrument is equipped with a Scott-type double-pass spray chamber. Optimization was carried out daily with a normal verification solution (10 ng/g, Rh, Mg, Pb, Ce, Ba). Two isotopes of mercury ($^{200}$Hg, $^{202}$Hg) were measured in each determination to obtain reliable results, in order to avoid possible interference. Data acquisition was undertaken by the ELAN software through a personal computer (IBM PS/2 Model 70). Peak areas were used as the analytical response and mercury concentrations were calculated after normalization of the data to the internal standard signal ($^{205}$Tl) followed by appropriate blank subtraction.

*Automated on-line hyphenated system:* An automated on-line hypenated D-CT-GC-QFAAS system [14–16, 21, 22] was used for mercury species analysis. This system combines five basic analytical steps (see figure 2): derivatization of mercury species to volatile forms; preconcentration by cryofocusing in liquid nitrogen; gas chromatographic separation during thermal desorption; detection by atomic absorption spectrometry; and data acquisition by a computer. All the steps are controlled through an electronic panel, which is programmed by a computer equipped with BORWIN software [23]. The set-up includes a peristaltic pump, 250-ml reaction vessel, two electronic Teflon-valves, a U-shaped Pyrex column (45 cm length × 5 mm id), Dewar bath, pneumatic pump, adjustable d.c. power supply, flow meter, T-shaped quartz furnace (light path length 20 cm, 1 cm id), atomic absorption spectrometer (Model 5000, Perkin-Elmer) and PC. The operation procedure is automatically and sequentially performed according to a programme previously defined in BORWIN software. First, $NaBH_4$ or $NaBEt_4$ solution is quantitatively transferred from the reaction flask to the reaction vessel by a peristaltic pump. The derivatization and purging steps take place in a 250-ml reaction vessel. The generated volatile Hg species are purged from the reaction vessel and trapped in the column, packed with 2.5 g of Chromosorb W HP (60–80 mesh) coated with 10% SP2100 (Supelco) and previously silanized with hexamethyldisilazane (Fluka). During cryofocusing, the column is immersed in a Dewar bath with liquid $N_2$ (−196°C) lifted by a pneumatic pump. In the desorption step, the column, wrapped with 0.5-mm-diameter Nichrome wire, is gradually heated by an adjustable power supply and the volatile mercury species successively elute in order of increasing molecular weight. The flow of the purging/stripping He gas is controlled by a flow meter. Atomization of mercury species occurs in a quartz furnace held at 800°C by means of an MHS-20 unit and detected by an atomic absorption spectrometer operated at 253.7 nm with a 0.7 nm slit-width. Data acquisition is finally undertaken by a chromatographic software run on a PC.

### Reagents

Analytical grade chemicals and Milli-Q water were used throughout (unless otherwise stated). A 0.1% mixed solution containing 1 ml of Triton X-100, 1 g of EDTA and 1 ml of $NH_4OH$ (25%), diluted to a final volume of 1 l with Milli-Q water, was prepared. An approximately 0.01% (m/v) solution of sodium tetraethylborate

($NaBEt_4$) was prepared in a glove-bag, filled with $N_2$, by dissolving the reagent in water. An approximately 4% (m/v) solution of $NaBH_4$ was prepared by dissolving the reagent in water. All vessels were first cleaned with RBS 50 detergent, thoroughly rinsed with tap water, soaked in a 10% $HNO_3$ solution for 24 h and finally rinsed with Milli-Q water before use.

*Standard solutions and certified reference materials*

A standard stock solution of 1000 μg ml$^{-1}$ of Hg(II) was prepared by dissolving mercury(II) chloride in 1% $HNO_3$, and that of 1000 μg ml$^{-1}$ of methylmercury by dissolving methylmercury chloride in methanol. All stock solutions were stored in a refrigerator and protected against light. Working standard solutions were prepared by appropriate dilution in water of the stock solutions and they were stored one week at maximum.

Four certified reference sediments, IAEA-356 (International Atomic Energy Agency of Monaco), PASC-1 (National Research Council of Canada), BCR S19 and CRM 580 (Community Bureau of Reference) and three biological reference materials, DORM-1 (Dogfish muscle) and TORT-1 (Lobster hepatopancreas) (National Research Council of Canada) and CRM 463 (Tuna fish muscle) (Community Bureau of Reference), were used to validate the proposed methods.

### Analytical procedures

*Analysis of sediments*

*Mercury species analysis:* A sample of approximately 1 g of homogenized dry sediment and 10 ml of acid solution were placed in an extraction tube and exposed to microwave irradiation at 60 W for 3 min. After irradiation, the sample solution was cooled to room temperature, transferred to a 15 ml tube and centrifuged at 5000 rpm for 5 min. The supernatant was poured into a 22-ml Pyrex vial with Teflon cap (Supelco) and finally stored in a refrigerator until analysis. A clean-up procedure was not necessary prior to the analysis after the aqueous phase ethylation method. An aliquot of 1 ml of the extract was analysed by means of the hyphenated Et-CT-GC-QFAAS system. Calibration was performed by the three-point standard-addition method to overcome possible matrix interferences and the sub-sample was subjected to triplicate analysis. Blanks were run after each triplicate analysis to check for the possible memory effects.

*Total mercury analysis:* A sample of approximately 0.25 g of homogenized dry sediment and 8 ml of concentrated nitric acid were placed in an extraction tube and exposed to microwave irradiation at 20 W for 5 min. After extraction, the sample was allowed to cool for about 5 min, followed by the addition of 2 ml of $H_2O_2$ and again digested at 20 W for further 5 min. After cooling, the extracts were diluted with Milli-Q water and finally stored in a refrigerator until analysis. An aliquot of 0.1 ml of the extract was added to a final solution (5 ml) containing 4 ml of a mixed Triton X-100 solution, which included 0.1% Triton X-100, 0.1% EDTA and 0.1% (v/v) ammonia solution, and an internal standard of thallium (100 ng). The resulting solution was analysed by FI-ICP-MS. The three-point standard-addition method in the same extract, and the addition of an internal standard were used to overcome matrix effects and instabilities of the instrument. Analyses were carried out in duplicate and measured using two isotopes of mercury ($^{200}Hg$, $^{202}Hg$). A blank test was prepared in each set of experiments to check for possible contamination during sample preparation and it was used to calculate the concentration of mercury after appropriate blank subtraction.

*Analysis of biotissues*

*Mercury species analysis:* A sample of 0.1–0.5 g of pulverized freeze-dried tissue and 5 ml of 25% TMAH alkaline solution were placed in an extraction tube and exposed to microwave irradiation at 60 W for 2 min. After irradiation, the sample solution was cooled to room temperature and then diluted with 5 ml of methanol. It was then transferred into a 22-ml Pyrex vial with a Teflon cap and stored in a refrigerator until analysis. It is not necessary to have a clean-up stage before analysis by hydride generation. Aliquots of 50–300 μl of the extract were directly analysed by the HG-CT-GC-QFAAS hyphenated system. The calibration, reproducibility analysis and blank test carried out in this case are similar to those already described for the analysis of sediments.

*FI-ICP-MS and automated on-Line D-CT-GC-QFAAS conditions:* The optimum parameters for FI-ICP-MS and automated on-line D-CT-GC-QFAAS operation are summarized in table 2.

### Results and discussion

*Optimum strategy for microwave-assisted extraction*

Extraction efficiency is the key to a successful microwave-assisted sample preparation for total and mercury species determination in environmental solution samples. In mercury speciation analysis, the extraction step must provide quantitative speciation of mercury species from the matrix without losses or contamination, and without changes in chemical forms; in total mercury analysis, mercury species must be not only completely liberated from the matrix, but also decomposed to Hg(II) without any loss and contamination. As a result, several variables, such as power applied, exposure time, and concentration and amount of extractant, must be carefully optimized when using an open focused microwave system. Once the optimum extraction agents have been chosen, the two most important variables influencing the extraction efficiency are power applied and exposure time. Figure 3 shows a generic view of extraction efficiency in the power setting versus irradiation time region. The domain of optimum efficiency is located in region B; in region C, above the boundary line of the upper limit, insufficient efficiency is achieved due to degradation or evaporation losses, because of a long time heating or intensive power setting. In region A, below the boundary line of the lower limit, incomplete dissolution or leaching also leads to

*Table 2. Optimum conditions for the automated on-line D-CT-GC-QFAAS system and FI-ICP-MS system for mercury speciation and total mercury analysis.*

| | *D-CT-GC-QFAAS* | | *FI-ICP-MS* | |
|---|---|---|---|---|
| | *Hydride* | *Ethylation* | | |
| *Derivatization* | | | *ICP-MS conditions* | |
| Derivatization solution | 5 ml of 4% m/v of $NaBH_4$ | 10 ml of 0.01% m/v of $NaBEt_4$ | Forward rf power | 1100 W |
| Solution pH | 0.2 ml of 12 mol $l^{-1}$ HCl | ≈4, 0.5 ml of 2 mol $dm^{-3}$ acetic/acetate buffer | Plasma gas flow rate | 15 l $min^{-1}$ |
| Reaction time | 0.5 min | 3 min | Auxiliary gas flow rate | 0.8 l $min^{-1}$ |
| | | | Nebulizer gas flow rate | 0.98 l $min^{-1}$ |
| *Cryogenic trapping* | | | | |
| GC column | U-shape glass tube, 45 cm length, 5 mm id | U-shaped glass tube, 45 cm length, 5 mm id | Sampler and skimmer cones | Nickel |
| GC phase | 10% SP-2100 on Chromosorb W-HP 60/80 mesh size | 10% SP-2100 on Chromosorb W-HP 60/80 mesh size | | |
| | | | *Data acquisition* | |
| Carrier gas | Helium (99.995%) | Helium (99.995%) | Scan mode | Peak hop transient |
| Pre-cooling duration | 1 min | 1 min | Dwell time | 100 ms |
| Purging duration | 4.5 min | 10 min | Sweeps per reading | 5 |
| Purging flow rate | 150 ml $min^{-1}$ | 150 ml $min^{-1}$ | Readings per replicate | 60 |
| | | | No. of replicates | 1 |
| *Desorption* | | | | |
| Stripping gas | Helium (99.995%) | Helium (99.995%) | Signal processing | Integrated |
| Stripping flow rate | 150 ml $min^{-1}$ | 150 ml $min^{-1}$ | Isotope measured | $^{202}Hg$, $^{200}Hg$ |
| Desorption voltage | 30 V | 30 V | Internal standard | $^{205}Tl$ |
| *Data acquisition* | | | | |
| Instrument | Perkin Elmer AAS 5000 | Perkin Elmer AAS 5100 | | |
| Wavelength | 253.7 nm | 253.7 nm | | |
| Quartz furnace temperature | 800°C | 800°C | | |
| Acquisition duration | 4 min | 5 min | | |

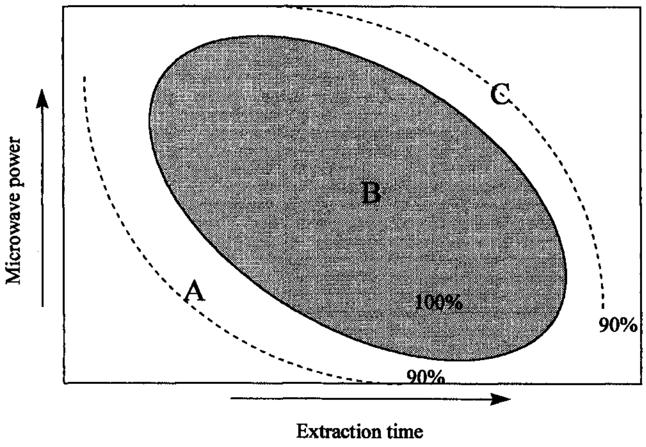

*Figure 3. Generic response surface of methylmercury recoveries in a microwave power versus extraction time.*

poor recoveries. The positions of both boundary lines may shift up and down depending on the strength of the extraction agent used and of the metal–matrix bonds. The energy focused on the sample can be calculated at each point in the matrix map of the power setting versus irradiation time according to: $Q = W \times T$ (where $Q$ is the energy output in cal, $W$ is power setting in cal/min and $T$ is the exposure time in min). Diagrams like that in figure 3 provide information about the optimum conditions required in each case to get quantitative recoveries during routine work, even though the energy needed to break the carbon–metal bonds remains unknown.

*Microwave-assisted extraction of sediments*

*Mercury speciation analysis:* The choice of extraction medium is the first step towards understanding the behaviour and extraction efficiency of methylmercury from sediments under mild microwave irradiation [14–16, 19, 20]. Acid solutions have commonly been used in the extraction of organomercury compounds from sediments [9, 24–28]. Thus, four different acid solutions, nitric (2 mol $dm^{-3}$), hydrochloric (2 mol $dm^{-3}$), sulphuric (1 mol $dm^{-3}$) and acetic acids (100%), were selected to check the stability of $MeHg^+$ and to investigate the $MeHg^+$ extraction efficiency from reference sediments in a microwave field. Each of the extractants was spiked with an amount of $MeHg^+$ and exposed to a microwave field during varying heating time at 60 W. The results obtained after up to 8 min heating show good stability of $MeHg^+$ in $HNO_3$ and HCl solutions, but only 80–90% of averaged $MeHg^+$ recoveries in $H_2SO_4$ and $CH_3COOH$ solvents. $MeHg^+$ losses are probably due to evaporation of extractant during vigorous heating. In another set of experiments, reference sediments suspended in the acid solutions mentioned above were exposed to microwaves at 60 W for 3 min. Quantitative recoveries were obtained by 2 M nitric and hydrochloric acids. Overall recoveries of about 85% and 55% for pure acetic acid and 1 mol $dm^{-3}$ sulphuric acid were observed, respectively. These low recoveries are mainly due to incomplete recovery from sediments by $CH_3COOH$ and partial adsorption on fine organic particles in the case of $H_2SO_4$. Additionally, interference problems were achieved in the determination step when analysing HCl, $H_2SO_4$ and $CH_3COOH$

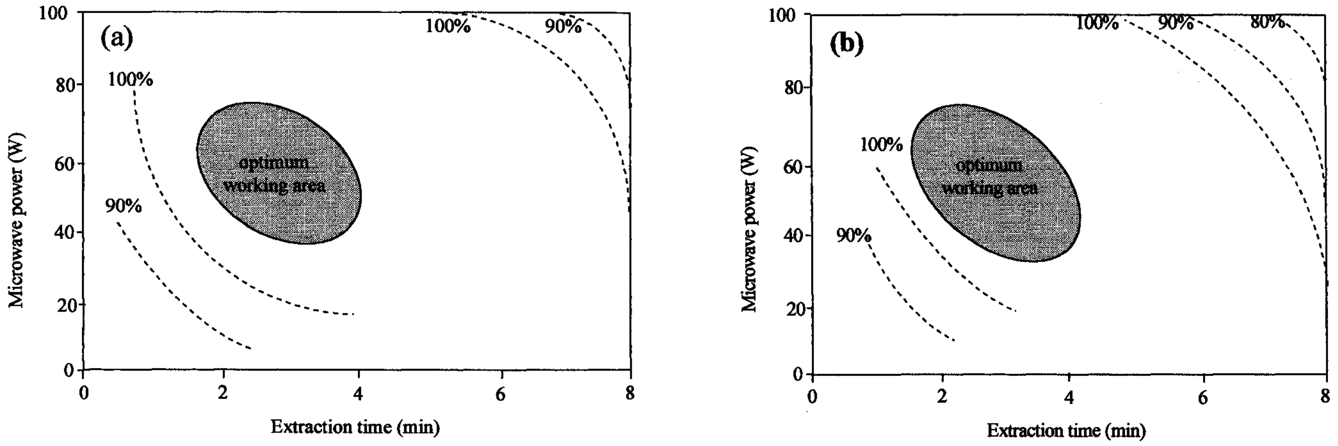

*Figure 4. Methylmercury recoveries from (a) BCR S19 and CRM N580 certified materials (extractant, 10 ml of 2 M $HNO_3$; sediment, 1 g) and (b) CRM 463 certified material (extractant, 5 ml of 25% TMAH; tissue, 0.2 g) as function of power applied versus time irradiation.*

leachates. This was not the case, however, for $HNO_3$ leachates. Taking into account all the facts mentioned above, nitric acid solution is an excellent extractant for methylmercury leaching from sediments, in terms of extraction efficiency and matrix interference. $MeHg^+$ microwave-assisted leaching from sediments with 2 mol dm$^{-3}$ $HNO_3$ was, therefore, optimized by constructing the corresponding response surface of power applied versus time irradiation using BCR S19 and CRM N580 reference materials [Figure 4(*a*)]. The optimum yields (100%) were obtained in 1–7 min in the 100–20 W range. Extreme conditions (longer time heating and/or higher power setting) result in evaporation losses or in rapid boiling out of the extractant, even though quantitative recovery may be achieved. Summarizing, 2–4 min heating versus 60–40 W power conditions are recommended as the optimum condition for microwave-assisted leaching of methylmercury from sediments using 2 mol dm$^{-3}$ $HNO_3$ as extractant. In a further recovery study, the effect of changing $HNO_3$ concentration was investigated. Quantitative and non-destructive $MeHg^+$ recovery was obtained by extraction with 2 mol dm$^{-3}$ up to 10 mol dm$^{-3}$ $HNO_3$ at 60 W for 3 min irradiation; 1 mol dm$^{-3}$ $HNO_3$ led to insufficient recovery and degradation of $MeHg^+$ for concentrated $HNO_3$.

*Analytical figures of merit:* A flow chart of the analytical procedure developed for methylmercury determination in sediments is shown in figure 5. It was validated by the analysis of three different certified reference sediments, BCR 19, CRM N580 and IAEA-356, using 2 and 6 mol dm$^{-3}$ $HNO_3$ solution as extractant. The results are in good agreement with the certified values, and reproducibility is similar to those obtained by conventional methods (table 3). The detection limit was calculated as 0.5 ng of $MeHg^+$ as Hg per g of dry sediment, with a linearity range from 0.5 to 100 ng of $MeHg^+$ as Hg. The comparison of the slope of the calibration curve with that obtained using aqueous $HNO_3$ spiked with $MeHg^+$ confirms that the analysis is not affected by matrix effects. No clean-up procedure is necessary, but the extract must be centrifuged after microwave irradiation prior to the analysis, in order to prevent readsorption on suspended matter. The chromatogram in figure 6(*a*) was obtained for the analysis of the reference CRM N580 sediment using 6 mol dm$^{-3}$ $HNO_3$ solution as extractant. The peak at 1.3 min corresponds to $Hg^0$ and is due to reduction of $Hg^{2+}$ during the determination step. High content of $Hg^{2+}$ in sediments is responsible for the appearance of such a peak for $Hg^0$.

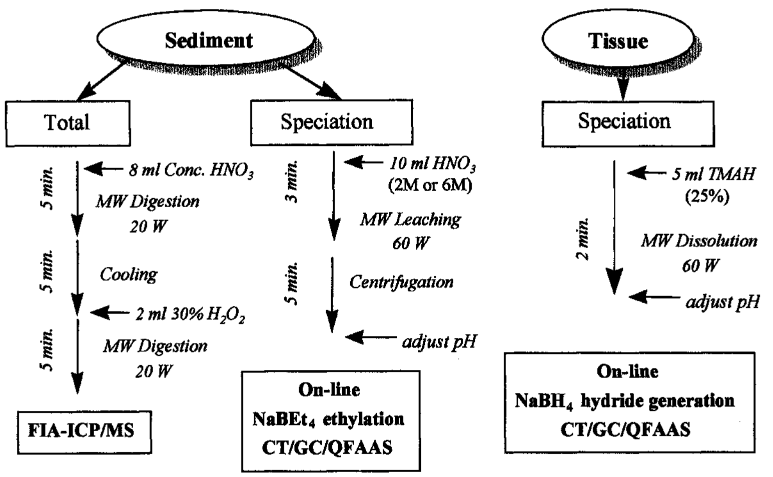

*Figure 5. Schematic analytical protocols for total and mercury speciation analysis in environmental samples.*

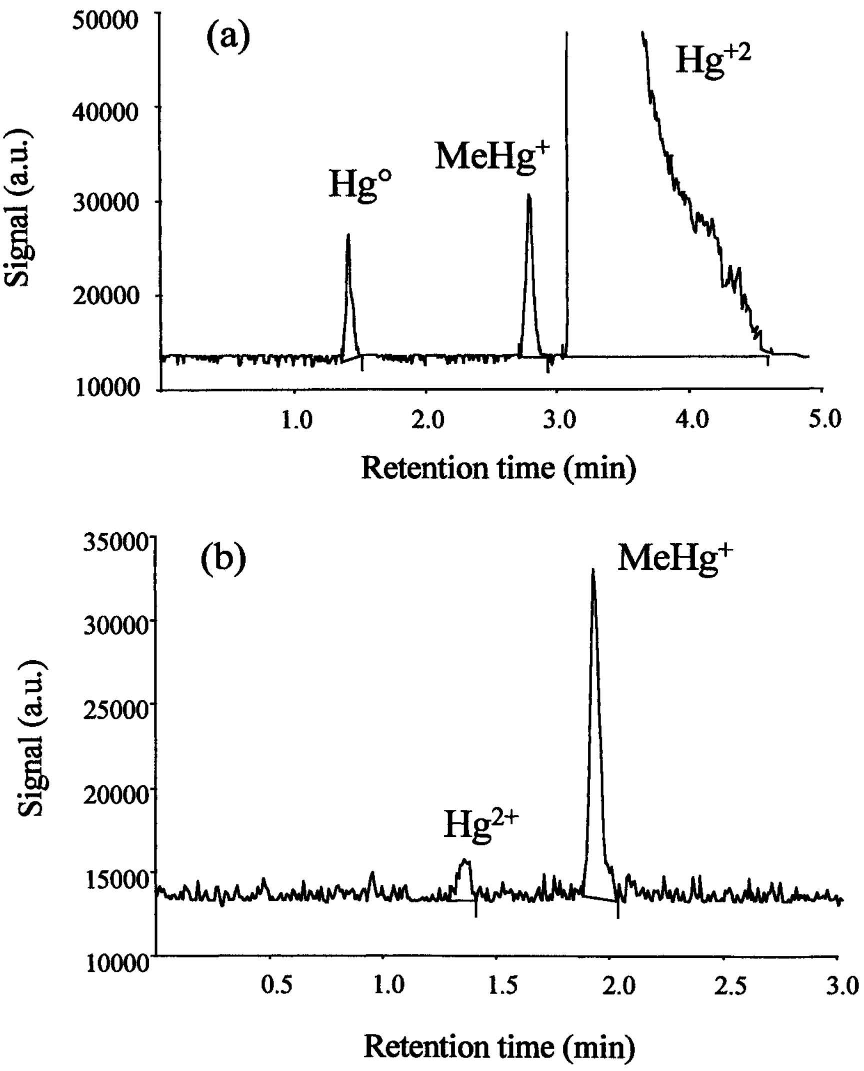

*Figure 6. Typical chromatograms of mercury species obtained by following the proposed procedures for (a) CRM N580 (0.5 ml of 6M $HNO_3$ extract) and (b) Dorm-1 (200 μl of 25% TMAH extract) certified materials*

*Table 3. Results for the determination of methylmercury in certified reference sediments using $HNO_3$ 2 mol $dm^{-3}$ or 6 mol $dm^{-3}$ as extractant in the microwave assisted extraction step.*

| *Sediment* | *Concentration of $MeHg^+$ ($ng\,g^{-1}$)*[1] *Determined*[2] | | *Certified* |
|---|---|---|---|
| | $HNO_3$ 2 mol $dm^{-3}$ | $HNO_3$ 6 mol $dm^{-3}$ | |
| BCR S19 | 51.9 ± 5.1 | 50.6 ± 4.5 | 53.1 ± 8.6 |
| CRM 580 | 79.6 ± 3.0 | 73.3 ± 5.7 | 75.4 ± 5.0 |
| IAEA-356 | 5.49 ± 0.72 | Not analysed | 5.87 ± 0.41 |

1. Calculated for dry mass.
2. Six independent experiments.

*Total mercury analysis:* The choice of the best extraction agent for microwave-assisted digestion and of the most appropriate analytical technique for the final determination are of crucial importance in total mercury analysis [29]. Mercury species are easily and strongly bound to organic matter, e.g. thiol groups, humic substances and amino acids. Thus, oxidizing agents such as concentrated $HNO_3$ and $H_2O_2$ solution were chosen to liberate the mercury species from the organic matrix and to fully oxidize them to Hg(II). The use of sulphuric acid as extractant is not recommended. The simple digestion procedure proposed here is as follows: the sediment (about 0.25 g) is decomposed by a two-step attack with (1) concentrated $HNO_3$ at 20 W power for 5 min, followed by cooling the mixture about 5 min; and (2) a subsequent extraction with 30% $H_2O_2$ at 20 W power for

*Table 4. Results for the determination of total mercury in certified reference sediments.*

| Sediment | Total mercury concentration ($\mu g\ g^{-1}$)[1] Determined[2] | Certified |
|---|---|---|
| BCR S19 | 95 ± 3 | 91.07 |
| IAEA-356 | 7.3 ± 0.1 | 7.62 |
| PACS-1 | 4.7 ± 0.3 | 4.57 ± 0.16 |

1. Calculated for dry mass.
2. Results given with 95% confidence interval.

5 min. Mild conditions prevent losses of mercury as $Hg^0$ during intensive microwave digestion in an open system. The results obtained after the analysis of the extracts by FI-ICP-MS showed the quantitatively and reproducibility of the digestion procedure described above. The use of an open-vessel microwave system facilitates the addition of the second reagent. No contamination was observed throughout all the experiments.

*Analytical figures of merit:* The simple analytical procedure illustrated in figure 5 takes only 15 min per sample. It can be shortened by automatic robust operation with an open focused microwave system when analysing samples in series. The method was validated by the analysis of three certified reference sediments, PASC-1, IAEA-356 and BCR S19, by FI-ICP-MS detection. The results obtained in the determination of total mercury are in good agreement with the certified values (table 4). Obtained recoveries ranged from 95 to 105% and the reproducibility was better than 10% in a mercury concentration range between 4 and 100 $\mu g\ g^{-1}$. The addition of Triton X-100 as surfactant and ETDA as complexing agent is necessary to obtain linear calibration curves in order to eliminate memory effects and improve efficiency during sample transport [29]. The use of an FI system prior to ICP-MS detection, standard addition method and complexation of mercury by EDTA improves reliability of the results, compared to conventional ICP-MS methods using direct calibration. Detection limits, calculated as three times the standard deviation of the blank divided by the slope of the calibration curve, are 10 pg $g^{-1}$ and 1.0 ng $g^{-1}$ for solutions and dry sediment samples, respectively.

### *Microwave-assisted extraction of biomaterials*

*Mercury speciation analysis:* To obtain quantitative recoveries of mercury species incorporated in the biological matrix, complete dissolution of the biological tissue is necessary [15, 16, 30]. Two candidate approaches to obtain good solubilization of biotissues are acid and alkaline hydrolysis procedures. In this study, five extraction agents, $HNO_3$, HCl, $CH_3COOH$, TMAH and methanolic-KOH solutions, were investigated to understand the stability and extraction efficiency of methylmercury in simple solutions and reference biomaterials. $MeHg^+$ stability in simple solutions was tested in the same way as for the sediments (see earlier). $MeHg^+$ was spiked in each extraction medium, followed by irradiation at a preset power for varying heating times. Quantitative recoveries were obtained for TMAH after 6 min of heating at 40 W power, whereas only 85–90% yields were observed for $CH_3COOH$ and methanolic-KOH, owing to volatile losses during long heating time. For concentrated $HNO_3$ and HCl $MeHg^+$, recoveries quickly decreased with heating time due to rapid breakdown and partial evaporation losses of $MeHg^+$ after 6 min of heating. Recoveries of $MeHg^+$ from reference biotissues at 40 W power for 4 min of irradiation significantly differ between solutions. Quantitative recoveries were systematically obtained with alkaline solutions such as 25% TMAH and methanolic KOH solutions. Poor recoveries were found, however, when using concentrated $HNO_3$ and HCl, owing to degradation and evaporation losses of $MeHg^+$ during microwave irradiation. As to pure acetic acid, lower yields of both methyl- and inorganic mercury were obtained using pure acetic acid as extractant due to incomplete dissolution of the tissue. Alkaline digestion was selected for $MeHg^+$ determination in biotissues. The potential chemical mechanisms of $MeHg^+$ extraction from organic matrix with acid or alkaline digestion have been previously discussed [15]. Owing to its higher extraction efficiency and lower solution volatility, compared with methanolic-KOH solution, 25% TMAH solution was chosen as extractant to investigate the optimum conditions for microwave-assisted digestion of biotissues. The power applied versus time irradiation response surface for $MeHg^+$ recoveries from CRM 463 biomaterial is shown in figure 4(*b*). Similar to the study of $MeHg^+$ optimization in sediments, the zone of quantitative yields (100%) is located at 1–6 min and 100–20 W. Special care must be taken, however, to avoid evaporation losses or rapid boiling out of the extractant in the case of long time heating and high power setting. As a result, irradiation for 2–4 min at 60–40 W using 25% TMAH solution as extractant is recommended as the optimum condition for microwave-assisted alkaline digestion of tissues in an open focused microwave system. In a further recovery study, the effect of various TMAH concentrations was investigated. Quantitative $MeHg^+$ recovery was obtained by extraction with 10–25% TMAH solution for 0.2–0.5 g of dry biotissue at 60 W for 2 min irradiation. These conditions allowed simultaneous quantitative extraction of methyl- and inorganic mercury from biomaterials.

*Analytical figures of merit:* The biotissue extract after microwave digestion can be analysed without any clean-up step [see figure 5]. The proposed analytical procedure was validated by analysing (HG-CT-QFAAS) three different reference biomaterials, CRM 463, DORM-1 and DORT-1, after 2 min/60 W microwave-assisted digestion of 0.1–0.5 g of tissue with 5 ml of 25% TMAH solution. The results obtained for methylmercury are in good agreement with the certified values, as shown in table 5. Inorganic mercury can also be simultaneously extracted and determined by this method. The sums of the concentrations of both mercury species present in the tissues also match certified total mercury content in the biotissues (table 5). A reproducibility of 4–10% was obtained in the determination of both mercury species. The detection limits for both $Hg^{+2}$ and $MeHg^+$ were calculated as 50 ng $g^{-1}$ for 0.2 g of pulverized dry sample and 0.05 ml of extract. Additionally, quantitative $MeHg^+$ recoveries were also

*Table 5. Results for the determination of methylmercury and inorganic mercury in certified reference biological tissues.*

| | Concentration ($\mu g\,g^{-1}$) as Hg[1] | | | | | |
|---|---|---|---|---|---|---|
| | Determined[2] | | | Certified | | |
| Sediment | $Hg^{2+}$ | $MeHg^{+}$ | Total Hg[3] | $Hg^{2+}$ | $MeHg^{+}$ | Total Hg[3] |
| CRM 463 | $0.235 \pm 0.030$ | $2.735 \pm 0.106$ | $2.970 \pm 0.110$ | $0.02 \pm 0.22$ | $2.83 \pm 0.15$ | $2.85 \pm 0.16$ |
| DORM-1 | $0.120 \pm 0.035$ | $0.728 \pm 0.028$ | $0.848 \pm 0.045$ | $0.067 \pm 0.095$ | $0.731 \pm 0.060$ | $0.798 \pm 0.074$ |
| TORT-1 | $0.184 \pm 0.024$ | $0.142 \pm 0.017$ | $0.326 \pm 0.029$ | $0.202 \pm 0.062$ | $0.128 \pm 0.014$ | $0.33 \pm 0.06$ |

1. Calculated for dry mass.
2. Three independent experiments.
3. Calculated as $Hg^{+2} + MeHg^{+}$.
4. Calculated as total Hg − $MeHg^{+}$.

observed for up to 1 g of dry biotissue using 5 ml of 25% TMAH solution as extractant. A typical chromatogram obtained for DORM-1 reference biotissue is shown in figure 6(*b*).

### Conclusions

Simple, rapid, efficient and quantitative sample leaching/digestion protocols based on a microwave-assisted technique have been developed for the determination of total and mercury species in environmental solid samples such as sediments and biomaterials. The use of an open focused microwave system offers reproducible and quantitative recovery of the analytes and keeps the organomercury species intact. The appropriate extractants, a combination of $HNO_3/H_2O_2$, $HNO_3$ and 25% TMAH solutions, were chosen for total and mercury species determination in sediments and biotissues after careful evaluation of the stability and extraction efficiency of methylmercury in a microwave field. Optimum extraction conditions of 2–4 min irradiation and 40–60 W power were selected for mercury speciation analysis following a matrix approach. Sample throughput can be controlled by instrumental analysis time, rather than by sample preparation step. A drastic reduction of time is achieved in sample preparation when microwave technology is used, compared to other currently available methods [15, 16, 31]. Microwave-assisted techniques for total and mercury speciation analysis offer advantages in terms of simplicity, reliability and analysis time and cost. This technique might be extended to provide similar sample preparation protocols for other metal and metalloids in environmental metrices [31–33].

### Acknowledgements

The authors wish to thank D. Mathé (Prolabo) for use of a commercial A301 microwave digestor; and Messrs Lobinski, Szpunar and Schmitt for their contribution to the microwave-assisted speciation-related research. C. M. Tseng acknowledges the Taiwan Government for his PhD grant. A. de Diego is grateful to the Spanish Government for his post-doctoral fellowship.

### References

1. Quevauviller, Ph., Maier, E. A. and Griepink, B., 1995, In *Quality Assurance for Environmental Analysis*, Eds Quevauviller, Ph., Maier, E. A., and Griepink, B. (Amsterdam: Elsevier).
2. Drabaek, I. and Iverfeldt, A., 1995, In *Quality Assurance for Environmental Analysis*, Eds, Quevauviller, Ph., Maier, E. A., and Griepink, B. (Amsterdam: Elsevier).
3. Bloom, N. S. J., 1989, *Canadian Journal of Fishery and Aquatic Science*, **46**, 1131.
4. Fischer, R., Rapsomanikis, S. and Andreae, M. O., 1993, *Analytical Chemistry*, **65**, 763.
5. Puk, R. and Weber, J. H., 1994, *Analytica Chimica Acta*, **292**, 175.
6. Horvat, M., May, K., Stoeppler, M. and Byrne, A. R., 1988, *Applied Organometallic Chemistry*, **2**, 515.
7. Horvat, M., Bloom, N. S. and Liang, L., 1993, *Analytica Chimica Acta*, 135.
8. Lee, Y. H., Munthe, J. and Iverfeldt, Å., 1994, *Applied Organometallic Chemistry*, **8**, 659.
9. May, K., Stoeppler, M. and Reisinger, K., 1987, *Toxicological and Environmental Chemistry*, **13**, 153.
10. Liang, L., Horvat, M., Cernichiari, E., Gelein, B. and Balogh, S., 1996, *Talanta*, **43**, 183.
11. Holak, W. J., 1995, *AOAC International*, **78**, 1124.
12. Emteborg, H., Bjorklund, E., Odman, F., Karlsson, L., Mathiasson, L., Frech, W. and Baxter, D., 1996, *Analyst*, **121**, 19.
13. Cleland, S. L., Olson, L. K., Caruso, J. A. and Carey, J. M., 1994, *Journal of Analytical and Atomic Spectrometry*, **9**, 975.
14. Tseng, C. M., De Diego, A., Martin, F. M. and Donard, O. F. X., 1997, *Journal of Analytical and Atomic Spectrometry*, **12**, 629.
15. Tseng, C. M., De Diego, A., Martin, F. M., Amouroux, D. and Donard, O. F. X., 1997, *Journal of Analytical and Atomic Spectrometry*, **12**, 743.
16. Tseng, C. M., Schmitt, V. O., De Diego, A. and Donard, O. F. X., 1998, submitted to *American Environmental Laboratory*.
17. Kingston, H. M. and Jassie, L. B., 1988, *Introduction to Microwave Sample Preparation* (Washington, D.C.: ASC).
18. Abu-Samra, A., Morris, J. S. and Koirtyohann, S. R., 1975, *Analytical Chemistry*, **47**, 1475.
19. Donard, O. F. X., Lalere, B., Martin, F. and Lobinski, R., 1995, *Analytical Chemistry*, **67**, 4250.
20. Lalere, B., Szpunar, J., Budzinski, H., Garrigues, P. and Donard, O. F. X., 1995, *Analyst*, **120**, 2665.
21. De Diego, A., Pecheyran, C., Tseng, C. M. and Donard, O. F. X., 1998, submitted.
22. Tseng, C. M., De Diego, A., Amouroux, D. and Donard, O. F. X., 1998, submitted.
23. *Logiciel Intuitif pour la Chromatographie*, version 1.2 (JMBS Developpements).
24. Hempel, M., Hintelmann, H. and Wilken, R.-D., 1992, *Analyst*, **117**, 669.
25. Hintelmann, H. and Wilken, R.-D., 1993, *Applied Organometallic Chemistry*, **7**, 173.
26. Quevauviller, P., Donard, O. F. X., Wasserman, J. C., Martin, F. M. and Schneider, J., 1992, *Applied Organometallic Chemistry*, **6**, 221.
27. Longbottom, J. E., Dressman, R. C. and Lichtenberg, J. C. J., 1973, *Journal of AOAC International*, **56**, 1297.

28. HORVAT, M., BYRNE, A. R. and MAY, K. 1990, *Talanta*, **37**, 207.
29. WOLLER, A., GARRAUD, H., MARTIN, F., DONARD, O. F. X. and FODOR, P., 1997, *Journal of Analytical and Atomic Spectrometry*, **12**, 53.
30. SZPUNAR, J., SCHMITT, V. O., LOBINSKI, R. and MONOD, J.-L., 1996, *Journal of Analytical and Atomic Spectrometry*, **11**, 193.
31. SCHMITT, V. O., DE DIEGO, A., COSNIER, A., TSENG, C. M., MOREAU, J. and DONARD, O. F. X., 1996, *Spectroscopy*, **13**, 99.
32. SCHMITT, V. O., SZPUNAR, J., DONARD, O. F. X. and LOBINSKI, R., 1997, *Canadian Journal of Scientific Spectroscopy*, **42**, 419.
33. SZPUNAR, J., SCHMITT, V. O., DONARD, O. F. X. and LOBINSKI, R., 1996, *Trends in Analytical Chemistry*, **15**, 181.

